# *rocF* affects the production of tetramethylpyrazine in fermented soybeans with *Bacillus subtilis* BJ3-2

**DOI:** 10.1186/s12896-022-00748-4

**Published:** 2022-07-04

**Authors:** Zhenli Liu, Yongjun Wu, Lincheng Zhang, Shuoqiu Tong, Jing Jin, Xian Gong, Jie Zhong

**Affiliations:** Key Laboratory of Plant Resource Conservation and Germplasm Innovation in Mountainous Region (Ministry of Education), Collaborative Innovation Center for Mountain Ecology & Agro-Bioengineering (CICMEAB), College of Life Sciences/Institute of Agro-Bioengineeringering, Guizhou University, Guiyang, 550025 Guizhou China

**Keywords:** *Bacillus subtilis*, Transcriptome sequencing, *rocF*, Fermented soybeans, TTMP

## Abstract

**Background:**

Tetramethylpyrazine (TTMP) is a flavoring additive that significantly contributes to the formation of flavor compounds in soybean-based fermented foods. Over recent years, the application of TTMP in the food industry and medicine has been widely investigated. In addition, several methods for the industrial-scale production of TTMP, including chemical and biological synthesis, have been proposed. However, there have been few reports on the synthesis of TTMP through amino acid metabolic flux. In this study, we investigated genetic alterations of arginine metabolic flux in solid-state fermentation (SSF) of soybeans with *Bacillus subtilis* (*B.subtilis)* BJ3-2 to enhance the TTMP yield.

**Results:**

SSF of soybeans with BJ3-2 exhibited a strong *Chi*-flavour (a special flavour of ammonia-containing smelly distinct from natto) at 37 °C and a prominent soy sauce-like aroma at 45 °C. Transcriptome sequencing and RT-qPCR verification showed that the *rocF* gene was highly expressed at 45 °C but not at 37 °C. Moreover, the fermented soybeans with BJ3-2Δ*rocF* (a *rocF* knockout strain in *B. subtilis* BJ3-2 were obtained by homologous recombination) at 45 °C for 72 h displayed a lighter color and a slightly decreased pH, while exhibiting a higher arginine content (increased by 14%) than that of BJ3-2. However, the ammonia content of fermented soybeans with BJ3-2Δ*rocF* was 43% lower than that of BJ3-2. Inversely, the NH_4_^+^ content in fermented soybeans with BJ3-2Δ*rocF* was increased by 28% (0.410 mg/kg). Notably, the TTMP content in fermented soybeans with BJ3-2Δ*rocF* and BJ3-2Δ*rocF* + Arg (treated with 0.05% arginine) were significantly increased by 8.6% (0.4617 mg/g) and 18.58% (0.504 mg/g) respectively than that of the BJ3-2.

**Conclusion:**

The present study provides valuable information for understanding the underlying mechanism during the TTMP formation process through arginine metabolic flux.

**Supplementary Information:**

The online version contains supplementary material available at 10.1186/s12896-022-00748-4.

## Background

Tetramethylpyrazine (TTMP) is a nitrogen-containing heterocyclic compound that contributes to the formation of many aromas and flavors compounds [1, 2]. TTMP is a member of the class of pyrazines detected in fermented foods, such as Chinese Baijius (Chinese liquor), vinegar, and soybean-based fermented foods [3, 4]. It is commonly used in the food industry as an important aromatic compound additive to enhance flavor [5, 6]. Recent data suggested that TTMP possesses diet therapy functions and may be used as an effective therapeutic drug to treat cardiovascular health and enhance cognition [7–9]. Over the recent years, the application of TTMP in the food industry and medicine has been widely investigated. Most studies have focused on developing methods for the industrial-scale production of TTMP, including chemical and biological synthesis [10, 11]. The biological synthesis of TTMP provides several advantages over chemical syntheses, considering it is an environmentally friendly and cost-effective process [5]. Moreover, pyrazines have been widely reported as important microbial secondary metabolite, which implies that microbial metabolism can produce the TTMP [12].

Fermented soybeans (also named Douchi) are one of the most popular foods in China due to the strong *Chi*-flavour (namely soybean-flavour [13], a special flavor of ammonia-containing smelly distinct from natto). *Bacillus subtilis* (*B. subtilis*) is one of the most important microorganisms for the fermentation process of Douchi. More than half-century ago, Kosuge et al*.* [14] first suggested that *B. subtilis* can synthesize TTMP. Moreover, a recent study proved that TTMP in Chinese liquor is mainly generated from *B. subtilis* under Micro-Oxygen conditions [6]. Thus, it has been proposed that the metabolic engineering of *B. subtilis* can enhance the production of TTMP*.* A high yield (2.5 g/kg) of TTMP was obtained using *B. subtilis* IFO 3013 inoculated soybeans and fermentation for 14 days [15]. However, industrial production still has several difficulties, such as low production [16] and a low conversion rate of precursor substances [17].

Over the years, genetic engineering has emerged as a powerful tool in studying the behavior of the production of TTMP [7, 18]. Meng et al. [5] showed that knocking the 2,3-butanediol dehydrogenase gene (*bdhA)* and adding 2,3-butanediol exogenously improve the production of TTMP in *B. subtilis*. Similarly, a mutant *bdhA* and glucose uptake protein (GlcU) were reported to affect the production of TTMP in *B. subtilis* 168 [6]. Moreover, two genes (*BDH1* and another *BDH2* coding 2,3-butanediol dehydrogenase) were deleted or overexpressed to improve the TTMP yield in *Saccharomyces cerevisiae* [19]. The TTMP can be generated from various precursors, including acetoin and ammonia [20]. Alterations of carbon flux into the acetoin biosynthesis pathway by blocking the degradation and competing pathways can enhance the TTMP yield [5]. Moreover, the TTMP has also been produced by condensing acetoin with NH_4_^+^ [21, 22]. However, there have been few reports on the synthesis of TTMP through amino acid metabolic flux. Recently, a new theory has been proposed suggesting that amino acids have close relationship with the production of volatile compounds such as pyrazines [23]. Various metabolites were generated by using *B. subtilis* during the fermentation of natto, such as peptone, peptides, amino acids, sugars, and organic acids, enhancing organoleptic of the final products [24]. Yet, their contribution to the organoleptic properties of the final products has not yet been investigated in detail.

In this study, we investigated genetic alterations of arginine metabolic flux in order to enhance the TTMP yield. The results showed that the fermented soybeans with BJ3-2Δ*rocF* had a significantly higher content of arginine and TTMP. Our findings provided detailed insights into the effects of *rocF* genes on the metabolism of arginine and TTMP in fermented soybeans with the *B. subtilis* BJ3-2.

## Results

### Summary of the sequencing data

**Fig. 1:**
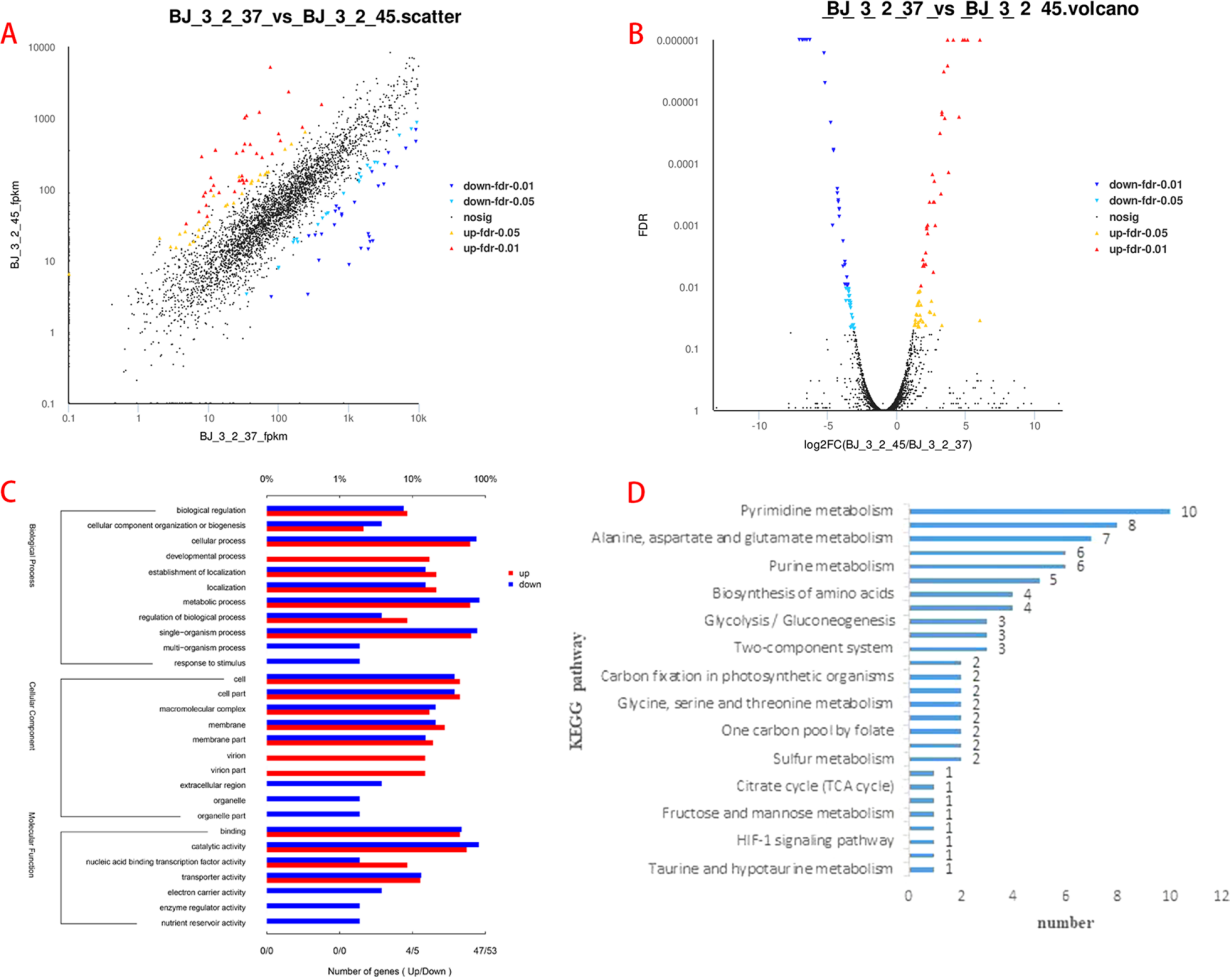
Analysis of DEGs. **A** Scatter map of DEGs; **B** Volcano map of DEGs; **C** GO enrichment analysis of DEGs; **D** KEGG pathway analysis of DE

The sensory evaluation of the fermented broth was performed by well-trained panelists on the three components that included soy sauce-like aroma, *Chi*-flavour, and ammonia; individual scores were added together to provide a total score (Table 1). According to the sensory evaluation, we found that the *Chi*-flavour of broth fermented with BJ3-2 at 37 °C was prominent, whereas the soy sauce-like aroma of broth fermented with BJ3-2 at 45 °C was prominent; the ammonia was similar at 37 °C and 45 °C (Table 1). Therefore, we performed RNA sequencing (RNA-seq) analysis of BJ3-2 at 37 °C and 45 °C. For the sequencing data, we found that the base content was evenly distributed, and the nucleotide distributions were within a reasonable range (Additional file 1: Fig. S1A, D). The quality and error rate of the samples were within a normal range (Additional file 1: Fig. S1B, C, E and F). After filtering the original data, checking the sequencing error rate, and verifying the distribution of the GC content, clean reads for follow-up analysis were also obtained. The Q20 and Q30 values of BJ3-2 at 37 °C were 98.5% and 95.93%, respectively, while they were 98.42% and 95.7% at 45 °C (Table 2). Next, gene expression analysis indicated that the gene expression levels in different samples were uniformly distributed in density diagrams (Additional file 1: Fig. S2). After normalizing the sequencing data, we constructed a scatter and volcano diagram (Fig. 1A, B). As shown, 3648 genes were identified, including 67 up-regulated DEGs and 56 down-regulated DEGs (*P*-value < 0.05 and |log_2_FC|≥ 1) (Table 3).

**Table 1:** Sensory evaluation of BJ3-2 at 37 °C and 45 °C

Strain	Soy sauce-like aroma (35)	*Chi*-flavor (35)	Ammonia (30)
BJ3-2 37 °C	18	32	22
BJ3-2 45 °C	33	19	20

**Table 2:** Summary of the sequencing data

Samples	Sequence	Base (bp)	Error rate (%)	Q20 (%)	Q30 (%)	GC (%)
BJ3-2 37 °C	13,969,028	1,863,773,158	0.0114	98.5	95.93	46.27
BJ3-2 45 °C	11,090,274	1,491,854,650	0.0116	98.42	95.7	47.2

**Table 3:** The DEGs of RNA-seq at 45 °C

Gene ID	BJ 3–2 37 °C	BJ 3–2 45 °C	log_2_FC (BJ 3–2 45 °C/BJ 3–2 37 °C)	*P*-value	FDR	Regulate
BSU13700	77.522	5185.634	6.06	1.56E-16	5.70E-13	Up
BSU17820	0	6.491	6.04	1.01E-03	3.50E-02	Up
BSU02160	8.142	289.265	5.13	1.68E-12	8.78E-10	Up
BSU12300	35.418	1082.334	4.93	4.74E-13	4.13E-10	Up
BSU11750	32.984	1008.288	4.93	2.23E-12	9.01E-10	Up
BSU39580	12.584	358.188	4.82	4.26E-10	1.04E-07	Up
BSU12310	12.634	355.103	4.8	3.10E-12	1.03E-09	Up
BSU14569	53.082	1210.986	4.51	1.17E-07	1.77E-05	Up
BSU00240	138.86	2344.553	4.08	1.60E-09	3.65E-07	Up
BSU02970	10.84	146.608	3.75	1.15E-06	1.40E-04	Up
BSU02170	33.435	435.053	3.7	1.81E-09	3.87E-07	Up
BSU11740	25.23	323.403	3.67	1.36E-08	2.62E-06	Up
BSU12090	31.566	349.892	3.47	1.28E-07	1.86E-05	Up
BSU36970	40.022	429.186	3.42	1.79E-08	3.26E-06	Up
BSU12320	9.033	90.74	3.31	1.02E-07	1.61E-05	Up
BSU17320	2.032	20.756	3.29	1.33E-03	4.20E-02	Up
BSU12340	11.731	114.535	3.28	8.88E-08	1.47E-05	Up
BSU11760	8.632	81.025	3.22	2.99E-06	3.11E-04	Up
BSU12330	10.765	97.552	3.17	2.41E-07	3.25E-05	Up
BSU13520	4.768	33.11	2.77	1.24E-05	1.01E-03	Up
BSU10710	12.019	81.937	2.76	7.47E-04	2.78E-02	Up
BSU36960	49.193	320.369	2.7	1.70E-06	1.94E-04	Up
BSU12360	7.415	48.837	2.7	4.64E-06	4.23E-04	Up
BSU06850	9.309	60.185	2.68	9.66E-05	5.78E-03	Up
BSU12350	14.441	90.526	2.64	4.64E-06	4.23E-04	Up
BSU37620	100.13	605.721	2.6	1.28E-06	1.51E-04	Up
BSU19670	3.952	23.716	2.56	3.82E-04	1.70E-02	Up
BSU33510	27.25	151.488	2.47	6.82E-04	2.59E-02	Up
BSU22860	4.692	25.493	2.42	6.57E-04	2.52E-02	Up
BSU00430	2.886	15.713	2.4	6.57E-04	2.52E-02	Up
BSU11240	30.261	155.485	2.36	4.58E-06	4.23E-04	Up
BSU11230	27.363	133.296	2.28	1.20E-05	9.97E-04	Up
BSU38450	59.481	281.155	2.24	1.84E-05	1.37E-03	Up
BSU00640	30.035	135.911	2.17	1.36E-05	1.06E-03	Up
BSU00650	107.821	484.02	2.17	1.50E-05	1.14E-03	Up
BSU07140	30.763	135.31	2.13	3.92E-05	2.80E-03	Up
BSU32850	3.438	15.261	2.12	1.31E-03	4.18E-02	Up
BSU30270	9.623	41.992	2.11	6.52E-05	4.32E-03	Up
BSU00830	81.386	319.295	1.97	6.36E-05	4.30E-03	Up
BSU28060	23.9	93.458	1.96	7.45E-05	4.60E-03	Up
BSU11250	32.143	123.455	1.94	7.38E-05	4.60E-03	Up
BSU00660	35.405	134.701	1.92	7.38E-05	4.60E-03	Up
BSU36550	417.195	1556.086	1.9	5.17E-05	3.63E-03	Up
BSU19050	7.277	27.08	1.88	1.08E-03	3.68E-02	Up
BSU30280	10.263	35.571	1.78	4.72E-04	1.96E-02	Up
BSU30290	8.444	28.629	1.75	9.44E-04	3.34E-02	Up
BSU38080	223.922	747.383	1.74	1.76E-04	9.59E-03	Up
BSU31060	17.602	58.817	1.73	2.31E-04	1.16E-02	Up
BSU25150	9.698	32.51	1.73	1.03E-03	3.53E-02	Up
BSU16740	18.631	61.488	1.72	7.91E-04	2.86E-02	Up
BSU32180	5.357	17.113	1.66	1.45E-03	4.43E-02	Up
BSU33500	42.669	132.323	1.63	2.43E-04	1.20E-02	Up
BSU37630	40.825	124.492	1.61	3.84E-04	1.70E-02	Up
BSU24580	6.11	18.56	1.59	1.11E-03	3.74E-02	Up
BSU05880	7.189	21.896	1.59	1.46E-03	4.45E-02	Up
BSU12069	6213.887	18553.429	1.58	2.52E-04	1.22E-02	Up
BSU11780	41.076	122.185	1.57	1.24E-03	4.13E-02	Up
BSU27310	29.935	87.795	1.55	4.25E-04	1.81E-02	Up
BSU12050	125.11	367.154	1.55	4.93E-04	1.98E-02	Up
BSU33950	56.006	161.841	1.53	4.66E-04	1.95E-02	Up
BSU00840	153.025	433.769	1.5	4.94E-04	1.98E-02	Up
BSU37460	22.934	64.67	1.49	7.93E-04	2.86E-02	Up
BSU02360	243.519	638.924	1.39	7.36E-04	2.77E-02	Up
BSU40950	51.79	134.426	1.37	1.30E-03	4.18E-02	Up
BSU02350	68.903	174.218	1.34	9.74E-04	3.42E-02	Up
BSU31050	64.173	161.213	1.33	1.13E-03	3.79E-02	Up
BSU11220	71.588	176.107	1.3	1.30E-03	4.18E-02	Up
BSU15510	1928.76	14.424	− 7.05	1.22E-13	2.22E-10	Down
BSU15520	2240.655	18.756	− 6.89	1.97E-13	2.40E-10	Down
BSU15500	1042.241	8.719	− 6.88	5.66E-13	4.13E-10	Down
BSU15530	2030.044	18.342	− 6.78	9.39E-13	5.71E-10	Down
BSU15550	1525.052	14.708	− 6.69	2.47E-12	9.01E-10	Down
BSU15560	2006.169	21.44	− 6.54	4.81E-12	1.46E-09	Down
BSU11990	94,545.251	1019.604	− 6.53	2.08E-12	9.01E-10	Down
BSU15540	1912.399	23.571	− 6.34	1.31E-11	3.69E-09	Down
BSU15490	268.461	3.331	− 6.29	2.98E-10	7.78E-08	Down
BSU37350	2,666,228.43	69,847.529	− 5.25	8.15E-09	1.65E-06	Down
BSU01620	379.143	10.008	− 5.23	2.88E-08	5.01E-06	Down
BSU37770	3233.527	117.411	− 4.78	1.57E-07	2.20E-05	Down
BSU01600	79.028	3.085	− 4.63	1.29E-05	1.02E-03	Down
BSU40330	4924.789	203.326	− 4.6	4.62E-07	6.02E-05	Down
BSU37760	2661.551	110.143	− 4.59	4.99E-07	6.28E-05	Down
BSU01630	639.373	31.384	− 4.34	2.85E-06	3.06E-04	Down
BSU40340	9319.344	465.809	− 4.32	2.35E-06	2.60E-04	Down
BSU18310	796.701	42.378	− 4.23	3.91E-06	3.97E-04	Down
BSU38560	1224.887	65.094	− 4.23	4.36E-06	4.23E-04	Down
BSU40320	6670.251	373.473	− 4.16	6.14E-06	5.47E-04	Down
BSU18300	795.359	44.677	− 4.15	6.46E-06	5.61E-04	Down
BSU15480	415.79	23.288	− 4.15	8.63E-06	7.32E-04	Down
BSU30560	3284.05	218.997	− 3.91	2.51E-05	1.83E-03	Down
BSU37260	331.894	22.608	− 3.87	7.70E-05	4.68E-03	Down
BSU37250	732.741	53.182	− 3.78	6.80E-05	4.43E-03	Down
BSU37780	9186.092	681.619	− 3.75	5.73E-05	3.94E-03	Down
BSU38570	655.683	48.934	− 3.74	1.60E-04	9.18E-03	Down
BSU33240	100.946	7.784	− 3.68	2.00E-04	1.07E-02	Down
BSU34370	102.087	7.975	− 3.66	3.79E-04	1.70E-02	Down
BSU01610	274.333	22.027	− 3.63	1.70E-04	9.52E-03	Down
BSU37790	2151.666	174.823	− 3.62	1.20E-04	7.04E-03	Down
BSU14700	3806.507	323.198	− 3.56	1.61E-04	9.18E-03	Down
BSU38580	682.143	57.556	− 3.56	1.72E-04	9.52E-03	Down
BSU33430	364.978	31.714	− 3.52	2.20E-04	1.13E-02	Down
BSU29020	7948.458	696.634	− 3.51	2.04E-04	1.08E-02	Down
BSU32130	1442.649	126.693	− 3.51	2.22E-04	1.13E-02	Down
BSU18340	521.503	45.961	− 3.5	2.12E-04	1.11E-02	Down
BSU29010	9626.346	860.824	− 3.48	2.55E-04	1.22E-02	Down
BSU37420	432.702	39.14	− 3.46	3.05E-04	1.43E-02	Down
BSU26900	2580.202	235.026	− 3.46	3.82E-04	1.70E-02	Down
BSU34360	506.423	46.282	− 3.45	3.92E-04	1.70E-02	Down
BSU18320	483.601	44.598	− 3.44	2.97E-04	1.41E-02	Down
BSU39380	2061.209	196.04	− 3.39	3.90E-04	1.70E-02	Down
BSU33440	188.58	18.049	− 3.38	5.00E-04	1.98E-02	Down
BSU06530	1375.189	132.495	− 3.37	4.26E-04	1.81E-02	Down
BSU06520	1500.562	147.454	− 3.35	4.81E-04	1.97E-02	Down
BSU05370	35.066	3.369	− 3.34	1.53E-03	4.61E-02	Down
BSU39370	2356.907	237.199	− 3.31	5.63E-04	2.21E-02	Down
BSU06460	851.54	86.678	− 3.29	1.44E-03	4.43E-02	Down
BSU37370	5365.257	567.502	− 3.24	7.88E-04	2.86E-02	Down
BSU18330	185.255	19.877	− 3.21	9.00E-04	3.22E-02	Down
BSU06590	161.895	17.886	− 3.17	1.36E-03	4.23E-02	Down
BSU26820	20,896.556	2374.192	− 3.14	1.30E-03	4.18E-02	Down
BSU13190	1899.828	215.47	− 3.14	1.32E-03	4.18E-02	Down
BSU06510	1424.683	164.735	− 3.11	1.61E-03	4.76E-02	Down
BSU37280	167.252	19.356	− 3.1	1.60E-03	4.76E-02	Down

### GO and KEGG enrichment analysis of the DEGs

To determine the functions of the DEGs, GO analysis revealed that these genes were based on their roles in biological processes (BPs), cellular components (CCs), and molecular functions (MFs). The results indicated that these DEGs were mainly enriched in 28 GO terms (11 BPs, 10 CCs, and 7 MFs; Q-value ≤ 1). For BPs, the exhaustive analysis showed that many DEGs were involved in cellular processes, metabolic processes, and single-organism processes. For CCs, most DEGs were enriched in the cell, cell part, and macromolecular complex. For MFs, many DEGs were involved in catalytic activity, binding, and transporter activity (Fig. 1C). Additionally, KEGG pathway analysis revealed that the dominant DEGs were enriched in pyrimidine metabolism and arginine and proline metabolism (Fig. 1D; Additional file 2: Table S4). Collectively, the results suggested that the expression of genes related to various pathways differed in BJ3-2 cultured at 37 °C and 45 °C.

### The expression of ***rocF*** in BJ3-2 incubated at 37 °C and 45 °C

**Fig. 2:**
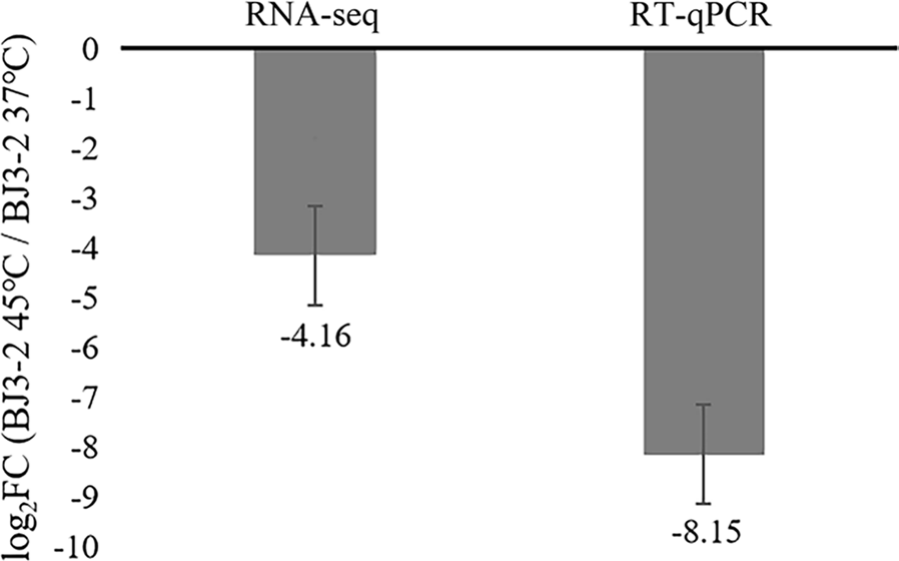
Verification of the expression of *rocF* by RT-qPCR

Through the analysis of RNA-seq data from BJ3-2 incubated at different temperatures, we observed that several genes were involved in arginine and proline metabolism (Table 4), which are closely associated with the products of TTMP in *B. subtilis* [21], thus suggesting that these genes may affect the products of TTMP. Among them, the expression of three genes (*fadM*, *argF,* and *argD*) was up-regulated at least 1 ~ fold (log_2_FC), and five genes (*rocD*, *rocF*, *sped*, *rocG,* and *rocA*) was down-regulated at least 3 ~ fold (log_2_FC) after incubation at 45 °C. Hampel et al*.* suggested that the knockdown of *rocD* affects the normal growth of microorganisms [25]. Interestingly, the expression of *rocF* was down-regulated by 4.16 ~ fold (log_2_FC) after incubation at 45 °C (Table 4). Therefore, we selected *rocF* for further investigation and its expression in BJ3-2 incubated at different temperatures by RT-qPCR. The results showed that the dissolution curve of *rocF* was a single peak, and the amplification curve had a high degree of coincidence (Additional file 1: Fig. S3). The expression of *rocF* was low at 45 °C and high at 37 °C. Notably, the RT-qPCR results were consistent with the RNA-seq data (Fig. 2).

**Table 4:** The DEGs in arginine and proline metabolism

Gene	Enzyme	log_2_FC (BJ3-2 45℃/BJ3-2 37℃)	*P*-value
*fadM*	Proline dehydrogenase	2.12	1.31E-03
*argF*	Ornithine carbamoyltransferase	1.94	7.38E-05
*argD*	Acetylornithine aminotransferase	1.3	1.30E-03
*rocD*	Ornithine aminotransferase	− 4.32	2.35E-06
*rocF*	Arginase	− 4.16	6.14E-06
*speD*	S-adenosylmethionine decarboxylase	− 3.48	2.55E-04
*rocG*	Glutamate dehydrogenase	− 3.62	1.20E-04
*rocA*	1-pyrroline-5-carboxylate dehydrogenase	− 3.75	5.73E-05

### Generation of the *rocF* knockout strain

To reveal the role of the *rocF* gene, the knockout vector pUC18-HLarm-*cm*-HRarm of *rocF* was constructed and transformed into *B. subtilis* BJ3-2 (Additional file 1: Fig. S4). The positive clones were verified by PCR (Additional file 1: Fig. S4E). Subsequently, the positive clones were sequenced, and the results indicated that the *rocF* were successfully replaced by a *cm* fragment in the pUC18-HLarm-*cm*-HRarm vector (Additional file 1: Fig. S5). These results demonstrated that *rocF* of *B. subtilis* BJ3-2 was successfully knocked out and denoted by BJ3-2Δ*rocF*.

### Characterization of the BJ3-2Δ*rocF* strain

**Fig. 3:**
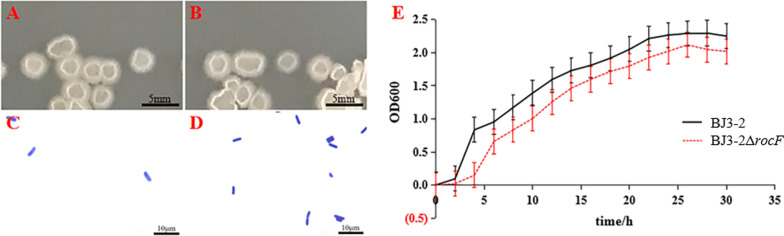
Colony morphology and growth curve. **A** Colony morphology of BJ3-2Δ*rocF*; **B** colony morphology of BJ3-2; **C** Gram staining of BJ3-2Δ*rocF*; **D** Gram staining of BJ3-2; **E** Growth curve of BJ3-2 and BJ3-2Δ*rocF*; Experiments were performed in triplicate; data are presented as means ± standard deviations (*n* = 3)

**Fig. 4:**
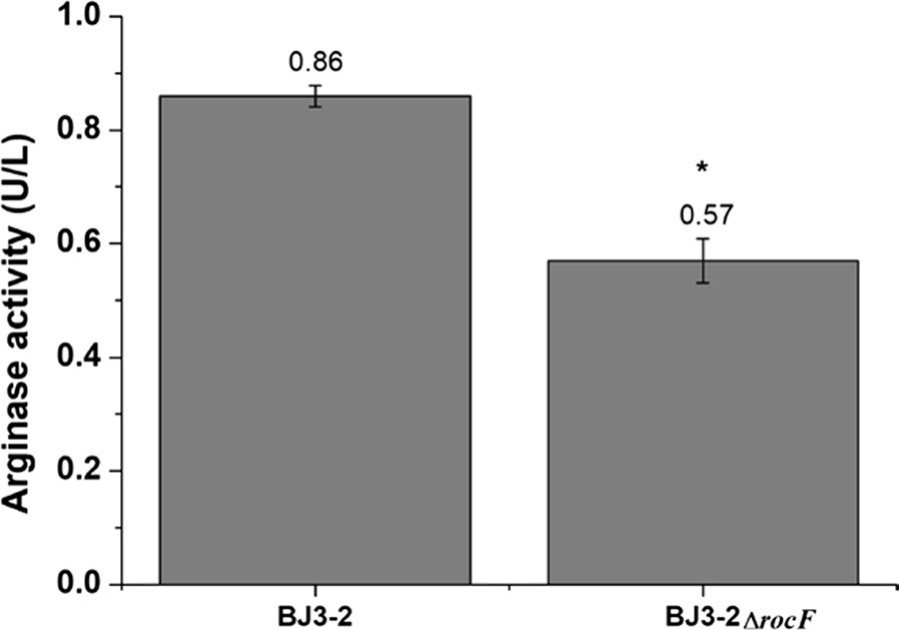
Activity of arginase in BJ3-2 and BJ3-2Δ*rocF***.** Each value represents the mean ± SE of three replicates. Asterisks indicate significant differences (*P* < 0.05) between BJ3-2 and BJ3-2Δ*rocF*

To investigate the growth ability of BJ3-2Δ*rocF*, the growth characteristics of BJ3-2Δ*rocF* were assessed. As expected, the color and shape (rough surface, irregular shape, and surrounding folds) of BJ3-2Δ*rocF* on the plate were similar to BJ3-2 (Fig. 3A, B). In addition, the microscopic morphology of BJ3-2Δ*rocF* was short and rod-shaped, similar to BJ3-2. Moreover, BJ3-2Δ*rocF* was also purple, similar to BJ3-2, as shown by Gram staining (Fig. 3C, D). The results indicated no significant differences in the morphology and color by Gram staining were observed between BJ3-2Δ*rocF* and BJ3-2. Correspondingly, almost the same results were observed for the growth curves of BJ3-2Δ*rocF* and BJ3-2 (Fig. 3E). However, the arginase activity was significantly inhibited in BJ3-2Δ*rocF* compared to BJ3-2 (Fig. 4). These results suggest that knockout of *rocF* only contributes to the inhibition of the arginase activity, but not change the growth rate of BJ3-2.

### Sensory evaluation of fermented soybeans

**Fig. 5:**
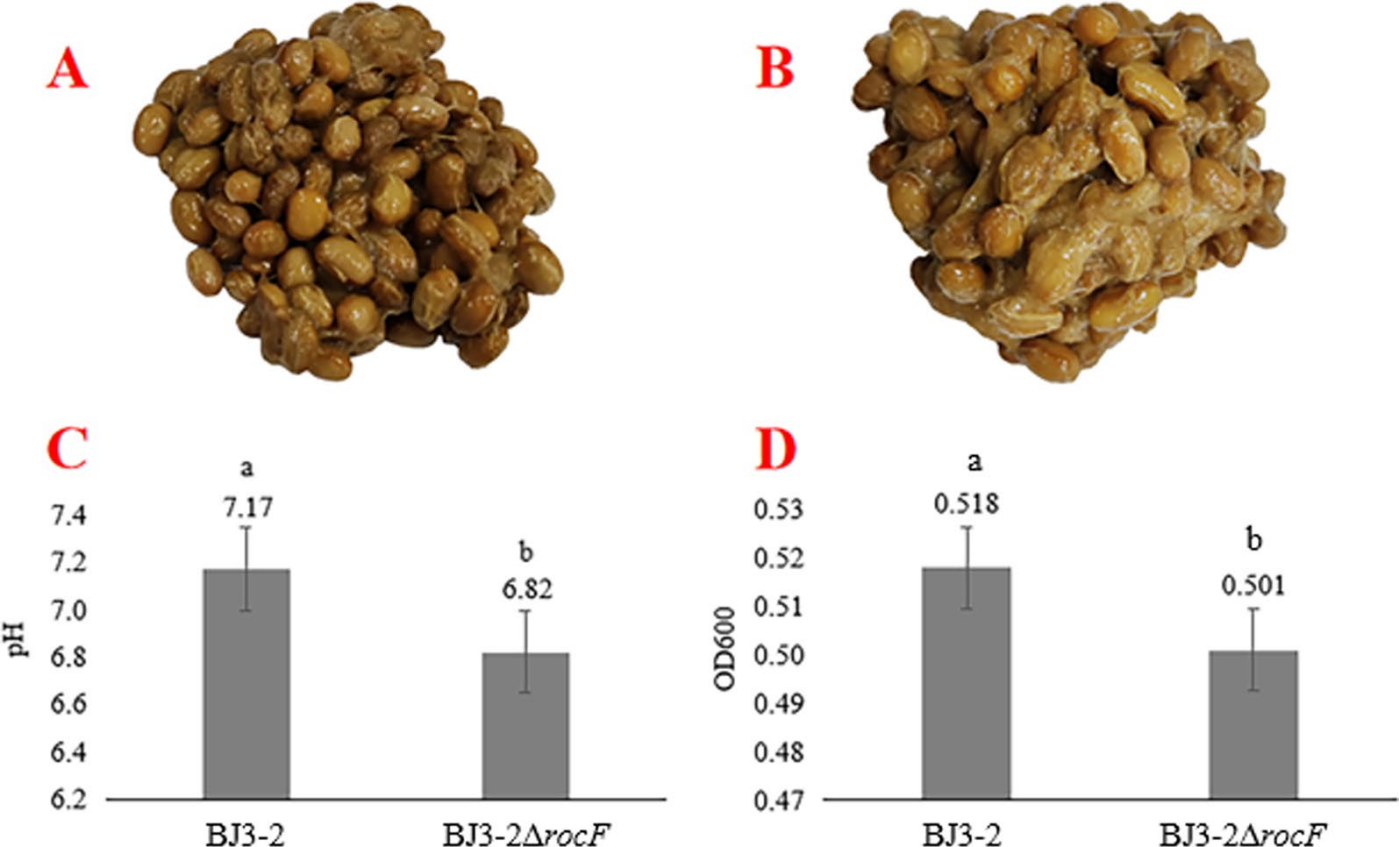
Fermented soybeans by BJ3-2 and BJ3-2Δ*rocF.* **A** Fermented soybeans by BJ3-2 at 45 °C; **B** fermented soybeans by BJ3-2Δ*rocF* at 45 °C; **C** pH values of fermented soybeans by BJ3-2 and BJ3-2Δ*rocF*; **D** OD600 of fermented soybeans by BJ3-2 and BJ3-2Δ*rocF.* In (**C**) and (**D**), values represent mean ± standard deviation obtained from independent triplicates. Different letters indicate significance difference amongst the various treatments (*P* < 0.05)

To assess the fermentation characteristics of BJ3-2Δ*rocF* in soybeans, the BJ3-2Δ*rocF* and BJ3-2 strains were inoculated with soybeans at 45 °C for 72 h. The sensory evaluation of the fermented soybeans was performed by well-trained panelists on five components that included two appearance components (color and stickiness), two aroma components (ammonia and soy sauce-like aroma), and texture, with the individual scores added together to provide a total score (Additional file 2: Table S5). According to the sensory evaluation, it was found that the soy sauce-like aroma of fermented soybeans with BJ3-2Δ*rocF* was prominent, whereas the ammonia was decreased (Additional file 2: Table S5). The degree of browning and viscosity were decreased in BJ3-2Δ*rocF* and BJ3-2Δ*rocF* + Arg (Fig. 5A, B) (Additional file 1: Fig. S6). Moreover, the pH of the fermented soybeans with BJ3-2Δ*rocF* was significantly lower than that of the fermented soybeans with BJ3-2 (Fig. 5C), indicating that knockout of *rocF* induced changes in the pH of the fermentation environment. In addition, the OD600 values of soybean slurries fermented with BJ3-2 and BJ3-2Δ*rocF* were 0.518 and 0.501, respectively (Fig. 5D), suggesting that the OD600 value of the soybean slurry fermented with BJ3-2Δ*rocF* was significantly reduced compared to that of BJ3-2.

### *rocF* decreased the production of ammonia

Following the sensory evaluation, we found that the ammonia of fermented soybeans with BJ3-2Δ*rocF* was decreased. Therefore, we further detected the ammonia content of the fermented soybeans by a gas detector. The results showed that the ammonia content of fermented soybeans with BJ3-2Δ*rocF* was 137.7 ppm, while the value was 241.6 ppm in BJ3-2, which was decreased by 43% (Table 5). The results indicated that the *rocF* gene affects the products of ammonia.

**Table 5:** Quantitative detection of arginine, NH_4_.^+^, and ammonia

Strain	Arginine content		NH_4_^+^ content		Ammonia content	
	Concentration (mg/mL)	AVG ± SD	Concentration (mg/kg)	AVG ± SD	Concentration (ppm)	AVG ± SD
BJ3-2	0.552	0.505 ± 0.038	0.288	0.297 ± 0.0074	245.8	241.6 ± 12.1
	0.502		0.296		253.8	
	0.460		0.306		225.1	
BJ3-2Δ*rocF*	0.599	0.590 ± 0.008	0.396	0.410 ± 0.0104^*^	135.1	137.7 ± 14.4^*^
	0.579		0.413		156.4	
	0.593		0.421		121.5	

* Significantly different at *P* < 0.05

### ***rocF*** enhanced the contents of arginine, NH_4_^+^, and TTMP in fermented soybeans

Indeed, arginine was converted into urea and ornithine through the urea cycle [26]. Therefore, the arginine content was measured by HPLC methodologies. The arginine content of fermented soybeans with BJ3-2Δ*rocF* was 0.590 mg/mL, which was dramatically increased compared to those fermented with BJ3-2 (Table 5). Subsequently, the content of NH_4_^+^ was detected by ion chromatography. A significant increase was obtained in fermented soybeans with BJ3-2Δ*rocF* (0.410 mg/kg) compared to those fermented with BJ3-2 (0.297 mg/kg) (Table 5). In addition, we also observed that the content of TTMP was increased by 8.6% in the fermented soybeans with BJ3-2Δ*rocF* (0.4617 mg/g) compared to those fermented with BJ3-2 (0.425 mg/g) (Table 6). Furthermore, a significantly increased content of TTMP in the BJ3-2Δ*rocF* treated with arginine were observed (Table 6), which indicated that *rocF* affects TTMP metabolic flow through the arginine pathway. However, the knockout of *rocF* resulted in slightly higher accumulations of the acetoin (Table 6). To sum up, these results indicated that the *rocF* gene affected the products of TTMP.

**Table 6:** Quantitative detection of acetoin and tetramethylpyrazine

Strain	Acetoin content		Tetramethylpyrazine content	
	Concentration (mg/g)	AVG ± SD	Concentration (mg/g)	AVG ± SD
BJ3-2	55.793	54.749 ± 0.702	0.418	0.425 ± 0.0046
	53.695		0.432	
	54.76		0.425	
BJ3-2Δ*rocF*	55.793	55.884 ± 0.061	0.446	0.4617 ± 0.0104^*^
	55.968		0.477	
	55.891		0.462	
BJ3-2Δ*rocF* + Arg	81.372	65.631 ± 10.493	0.492	0.504 ± 0.0177^*^
	57.571		0.488	
	57.953		0.530	

* Significantly different at *P* < 0.05

## Discussion

*B. subtilis* is an aerobic, Gram-positive soil bacterium widely used in the food industry [6, 27, 28]. *B. subtilis* BJ3-2, used in this study, was isolated from fermented soybeans [29]. Fermented broth with BJ3-2 exhibited a prominent soy sauce-like aroma at 45 °C (Table 1). Furthermore, RNA-seq was performed on BJ3-2 (at 37 °C and 45 °C). The dominant DEGs were enriched in pyrimidine metabolism and arginine and proline metabolism (Additional file 2: Table S4). Most DEGs, including *carA*, *pyrAa*, *pyrC*, *pyrDI*, *pyrDII, carB* (BSU11240), *pyrE*, *carB* (BSU15520), *pyrF*, and *pyrB*, were involved in pyrimidine metabolism. The deficiency of each gene affects pyrimidine biosynthesis and ultimately leads to pyrimidine-deficient strains [30–32]. Moreover, eight DEGs (*fadM*, *argF*, *argD*, *rocD*, *rocF*, *speD*, *rocG,* and *rocA*) were enriched in arginine and proline metabolism (Table 4). Arginine metabolism is closely related to the products of TTMP, which contributes to the soy sauce-like aroma [33]. Among them, the expression of three genes (*fadM*, *argF,* and *argD*) was up-regulated at least 1 ~ fold (log_2_FC), and five genes (*rocD*, *rocF*, *sped*, *rocG,* and *rocA*) were down-regulated at least 3 ~ fold (log_2_FC) after incubation at 45 °C. The *rocD* was the most significantly down-regulated gene (4.32 ~ fold), while the knockdown of *rocD* resulted in arginine-deficient strains [25]. Interestingly, the *rocF* of BJ3-2 was down-regulated 4.16 ~ fold at 45 °C compared to 37 °C (Table 4), indicating its potential function in producing the soy sauce-like aroma.

Recently, studies have found that the deletion of key genes in the microorganisms may harm strain growth. For example, the knockout of the *surfactin synthetase* (*srf*) gene seems not to be favorable for strain growth in *B. subtilis* PB2-L [34]. However, in this study, the growth of the BJ3-2Δ*rocF* strain showed no significant changes in color and morphology, and growth curves compared to BJ3-2 (Fig. 3), suggesting the knockout of the *rocF* gene does not affect the normal growth of BJ3-2. However, the arginase activity was significantly inhibited in BJ3-2Δ*rocF* (Fig. 4), suggesting knockout of *rocF* only contributes to the inhibition of the arginase activity, but not change the growth characteristics of BJ3-2. Moreover, nonenzymatic browning is important in evaluating the Maillard reaction products (MRPs) [35, 36], which is affected by the reaction time, temperature, pH, solvent, and other conditions [37–40]. In the final stage of the Maillard reaction, the browning intensity is enhanced by increasing the initial pH value and other conditions [41]. In our study, the fermented soybeans with BJ3-2Δ*rocF* exhibited a lighter color and a slightly decreased pH (Fig. 5). These results are consistent with a number of previous studies on the Maillard reaction.

**Fig. 6:**
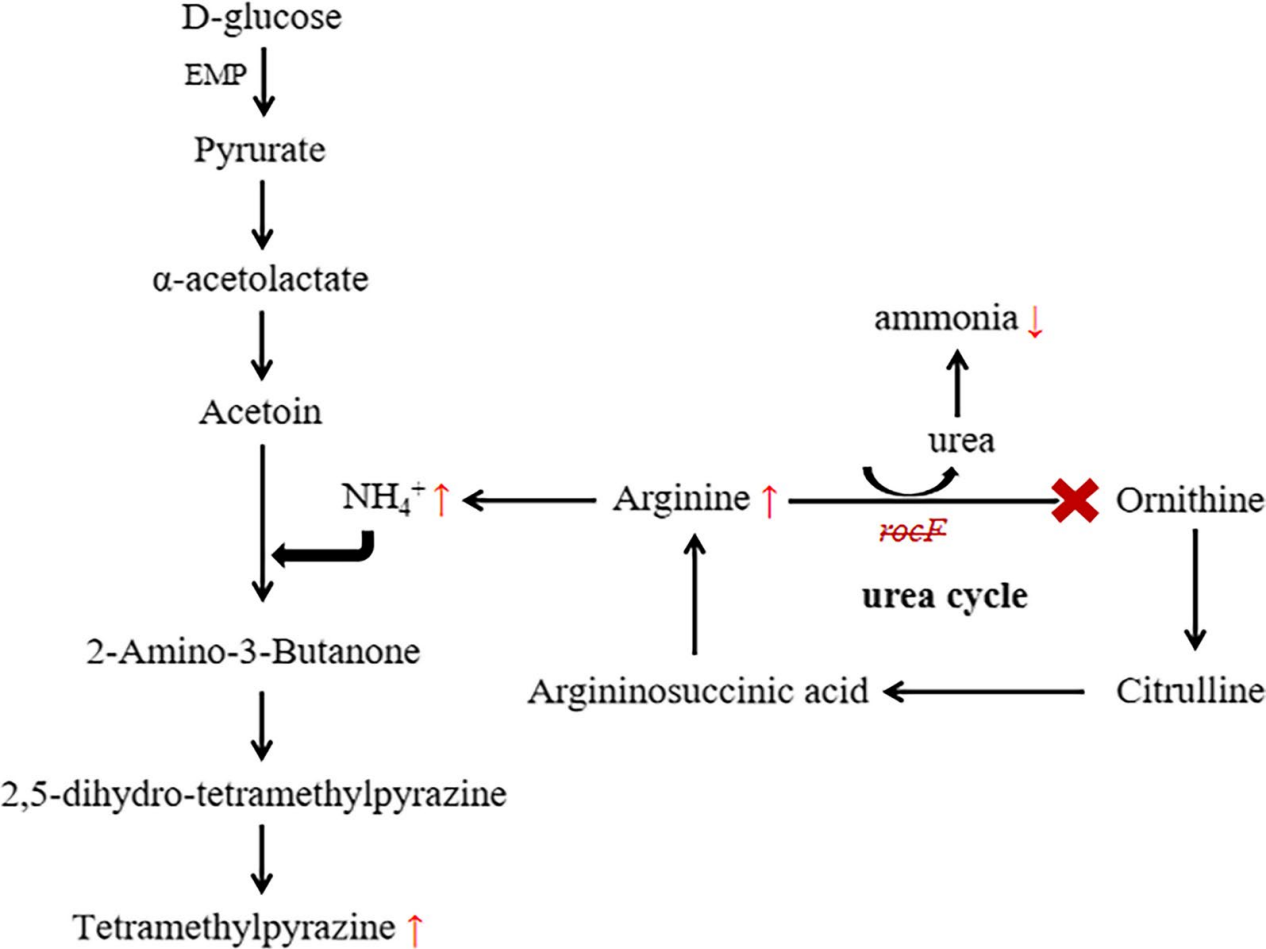
The metabolic pathways of arginine and TTMP

Zhao et al.reported a strong relationship between the amino acids and pyrazines [23]. In our study, the arginine content in fermented soybeans with BJ3-2Δ*rocF* was dramatically increased compared with BJ3-2 (Table 5), indicating the *rocF* gene affects arginine metabolism. In addition, we found that deletion of *rocF* gene changes the expression of arginine and proline metabolic pathway genes (Additional file 2: Table S4). Therefore, we speculated that *rocF* participates in the arginine and proline metabolic pathway and may convert arginine into urea and ornithine in the urea cycle (Fig. 6). Moreover, Rajini et al.reported that the production of TTMP is closely associated with amino acid metabolism and glycolytic pathways [12]. The amino acid metabolic pathway mainly provides a nitrogen source (NH_3_ or NH_4_^+^), and the glycolytic pathway mainly provides the precursor compound (acetoin) for the production of TTMP [21]. In our study, the knockout of the *rocF* gene dramatically increased the contents of NH_4_^+^ and TTMP in fermented soybeans (Table 5, 6). Furthermore, a significantly increasing of TTMP content was also observed in the BJ3-2Δ*rocF* treated with arginine (Table 6). Additionaly, we detected the ammonia of the fermented soybeans, and the results showed that the ammonia content was significantly reduced in BJ3-2Δ*rocF*, while one unexpected finding was the extent to which the soy sauce-like aroma was prominent (Additional file 2: Table S5). Thus, it is suggested that arginine could not be decomposed into urea and ornithine through the urea cycle in BJ3-2Δ*rocF,* which causes a reduction in the urea content. In accordance with the present results, previous studies have demonstrated that urea was degraded to ammonia by urease [42]. Collectively, we proposed a model to elucidate the potential function of *rocF* in changed arginine metabolic flux to enhance the TTMP yield (Fig. 6).

## Conclusion

In this study, a comparative metabolite and transcription profiling of BJ3-2 strain at 37 °C and 45 °C showed transcriptional changes in arginine and proline metabolism, which are closely associated with the products of TTMP in *B. subtilis*. In addition, the function of *rocF* gene was further studied, and *rocF* knockout mutant (BJ3-2△*rocF*) was generated using homologous recombination. The results indicated that the knockout of *rocF* affects the contents of arginine, ammonia, NH_4_^+^, and TTMP in fermented soybeans. Therefore, our data provide new light into the understanding of the production of TTMP in *B. subtilis*.

## Methods

### Strains and vectors

The strain (*B. subtilis* BJ3-2) was isolated from fermented soybeans (Patent No. 201110023795.4; https://www.patentstar.com.cn/.) [29]. The genome sequences of *B. subtilis* BJ3-2 have been submitted to NCBI (GI: CP025941). *Escherichia coli* DH5α and pUC18 vectors were purchased from TaKaRa (Dalian, China). The pHT01cas9-p43 vector was acquired from Biomics Biotechnology (Jiangsu, China).

### The collection of *B. subtilis* BJ3-2

A *B. subtilis* BJ3-2 single colony was cultured in 5 mL of liquid Luria–Bertani (LB) medium (10 g/L of tryptone, 5 g/L of yeast extract, and 5 g/L NaCl) and grown at 37 °C for 12 h under shaking at 180 rpm. The above bacterial cultures (3 mL) were reinoculated in 300 mL of liquid LB medium (1%) and divided into two groups, one of which was incubated at 37 °C, and the other was incubated at 45 °C, for 12 h under shaking at 180 rpm. Then, the cultures of the exponential growth phase were collected in a 50 mL sterile centrifuge tube and centrifuged at 5,000 g for 8 min. The sedimentation for each treatment was collected after discarding the supernatant, and the procedure was repeated three times.

### Total RNA extraction

After the collection of BJ3-2, total RNA was extracted using TRIzol reagent (Invitrogen, Carlsbad, CA, USA) by following the manufacturer's protocol. The concentration and purity of the total RNA were assessed using a Nanodrop ND-2000 spectrophotometer (Thermo Fisher Scientific, Wilmington, DE, USA). The RNA integrity values (RIN) of the total RNA were evaluated using an agarose gel and an Agilent 2100 bioanalyzer (Agilent, Palo Alto, CA, USA).

### RNA sequencing

The samples for each treatment were collected at least three times and pooled for RNA-seq analysis. Total RNA was extracted as described above. RNA-seq was performed by the Majorbio Technology Co., Ltd. (Shanghai, China). The 16S rRNA was used as an internal standard. Sequences of the raw data containing a small number of reads with sequencing adaptors or low-quality sequences were filtered. For accurate sequencing, we adopted a sequencing error rate distribution examination to establish the quality of sequencing data. Then, the differentially expressed genes (DEGs) were screened using the following conditions: *P*-value < 0.05 and |log_2_fold change (FC) |≥ 1. GO (Gene Ontology, http://www.geneontology.org/) enrichment analysis was performed to determine the DEGs that were common at 37 °C and 45 °C with opposite regulatory patterns with a threshold of *P* < 0.05. Proteins were filtered based on their grouping into biological processes (BP), cellular components (CC), and molecular function (MF). KEGG(Kyoto Encyclopedia of Genes and Genomes, http://www.genome.jp/kegg/) pathway enrichment analysis was performed for each DEG [43]. The raw reads and processed RNA-seq data in this work have been deposited in the NCBI Gene Expression Omnibus (GEO) database under record number GSE166082.

### Reverse transcription-quantitative real-time PCR (RT-qPCR)

To detect *rocF* gene expression, the total RNA extracted above was used to synthesize the first chain cDNA using the StarScript II First-strand cDNA Synthesis Mix with gDNA Remover Kit (GenStar, Beijing) following the manufacturer’s recommendations. Then, the cDNA was diluted to 200 ng/µl, and RT-qPCR was performed using a CFX96 Touch PCR instrument (Bio-Rad, USA). The composition of the reaction mixture and conditions were the same as previously described in Zhang’s study [44]. Each RT-qPCR analysis experiment was performed three times, and the primers used in this study are listed in Table S1. 16S rRNA was used as a reference gene for expression analysis.

### Homologous recombination knockout vector construction and transformation

The HLarm and HRarm correspond to the homologous left and right arms of *rocF*, whereas *cm* corresponds to the chloramphenicol gene. DEDP is a double-exchange detection primer (Additional file 2: Table S1) for assessing the success of the transformation.

The HLarm and HRarm of *rocF* were amplified from the *B. subtilis* BJ3-2 genome. The *cm* was amplified from the pHT01cas9-p43 vector. The primers used for amplification are listed in Table S1. HLarm, *cm,* and HRarm were double-digested with the restriction endonucleases *Sac* I and *Bam*H I, *Bam*H I and *Xba* I, and *Xba* I and *Hin*d III, respectively. The digested fragments of HLarm, *cm,* and HRarm were sequentially connected to the pUC18 vector. Finally, the homologous recombination knockout vector pUC18-HLarm-*cm*-HRarm was constructed. The recombinant plasmid was then transformed to *B. subtilis* BJ3-2 according to the description in the literature [45, 46]. The transformants were verified by PCR using DEDP primers (Additional file 2: Table S1). The genomic DNA of the transformants was extracted using the Bacterial DNA Kit (OMEGA, USA) following the manufacturer’s recommendations and sequenced by the Sangon Biotech Co., Ltd. (Shanghai, China).

### Enzyme determination assays

For the arginase determination assays, 0.1 g of BJ3-2 and BJ3-2Δ*rocF* bacteria were collected into a 1.5 mL centrifuge tube, sterile PBS (pH = 7.2) was added and broken by ultrasound in an ice bath and subjected to centrifugation (8,000 g for 5 min) to obtain the supernatant. The supernatant was transferred to a new tube used for the further assay. The arginase activity in BJ3-2 and BJ3-2Δ*rocF* was analyzed using the MEIMIAN reagent kit (Shanghai, China) and MULTISKAN GO (Thermo Fisher Scientific, Wilmington, DE, USA) according to the manufacturer’s instructions. A series of 2.5U/L, 5 U/L, 10 U/L, 20 U/L and 40 U/L concentrations were used to generate a standard curve. Three independent experiments were performed for each sample. The assays were done at least in triplicate.

### Fermentation experiments

BJ3-2Δ*rocF* and BJ3-2 were inoculated in 5 mL of liquid LB medium and subsequently incubated at 37 °C with shaking at 180 rpm for 12 h. The soybeans were autoclaved at 121 °C for 20 min. The above bacterial suspensions (OD600 = 0.465) of BJ3-2Δ*rocF* and BJ3-2 were inoculated in autoclaved soybeans (1%, v/m) and fermented at 45 °C for 72 h.

### Sensory evaluation

To assess the fermentation characteristics, the BJ3-2 strain was inoculated in a liquid LB medium and incubated at 37 °C and 45 °C for 72 h. The sensory analysis of the fermented broth was evaluated by 10 trained expert sensory panelists. The sensory score was a total of 3 indicators, including soy sauce-like aroma, *Chi*-flavour and ammonia (Additional file 2: Table S2). The sensory analysis of fermented soybeans by different strains was evaluated by 10 trained expert sensory panelists. The sensory score was a total of 5 indicators, including color, stickiness, ammonia, soy sauce-like aroma and texture (Additional file 2: Table S3).

### pH measurement

The pH test was performed according to the Chinese national standard (GB5009.237–2016). Briefly, slurry was created from the fermented soybeans (10 g) using ultrapure water (50 mL), and the pH of the slurry was measured using a standard laboratory pH meter (pHS-3C). Experiments were performed in triplicate.

### OD600 detection for the color of fermented soybeans

Fermented soybeans (20 g) were weighed and placed into a beaker with 100 mL of ultrapure water. The viscoid was cleaned, and the excess water of the soybeans was absorbed. Five grams of cleaned soybeans were placed in a mortar and ground into the homogenate with a pestle. Then, 50 mL of ultrapure water was added and mixed in the above homogenate. The mixture was well stirred and centrifuged at 8,000 g for 10 min. The absorbance of the supernatant fluid was measured at OD600. Experiments were conducted in three times.

### Ammonia contents of fermented soybeans

The ammonia content of the fermented soybeans was detected by a gas detector (WOST Co., Ltd., Shenzhen, China) following the manufacturer’s recommendations.

### Acetoin and TTMP contents of fermented soybeans

The concentrations of acetoin and TTMP were analyzed by HPLC. In brief, 10 g fermented soybean with BJ3-2, BJ3-2Δ*rocF* and BJ3-2Δ*rocF* + Arg (treated with 0.05% arginine) were dissolved in 30 mL 60% ethanol with 0.1 g CaCl_2_ and sonicated for 30 min at 25 °C (100 W, 20 kHz), and then centrifuged at 9000 rpm/min for 10 min at 4 °C. The supernatant was filtered by a 0.22 μm membrane before injection. The analysis was performed using Agilent LC1260 liquid chromatography (LC) system (California, USA), which was equipped with a flame ionization detector, a capillary column of Shim-pack GIST C18-AQ 5 μm (4.6 I.D. × 250 mm, SHIMADZU, Japan). Water with a trifluoroacetate concentration of 0.05% was mixed with methanol in a ratio of 7:3 (v/v) and used as the mobile phase at a flow rate of 0.7 mL/min. The column oven was kept at 30 °C for 5 min, then programmed to 220 °C with a stepwise increase of 30 °C /min and maintained at 220 °C for 3 min. The injection volume was 5 μL.

### Arginine content of fermented broth

BJ3-2Δ*rocF* and BJ3-2 were inoculated in liquid LB medium (1% v/v) and fermented at 45 °C for 72 h. Subsequently, 2 mL of fermented broth was collected and centrifuged at 10,000 g for 2 min. Then, the supernatant was collected and stored at 4 °C. The arginine content was analyzed using a Waters 1525 high-performance liquid chromatography (HPLC) system equipped with an automatic injector and an ultraviolet detector (UV) at 210 nm (Waters Corp., Milford, USA), according to the description in the literature [47]. Briefly, all fermented samples were treated with potassium ferrocyanide (106 g/L) and zinc sulfate (300 g/L) and then centrifuged at 2,000 g for 8 min. Next, the supernatant was filtered through a 0.22 μm filter membrane prior to injection for HPLC analysis. Each injection volume was set to 10 µl. Chromatographic separation was achieved using a Waters Atlantis C18 column (5 μm, 4.6 × 150 mm) (Waters, USA) at 30 °C. The mobile phase of HPLC was 20 mmol/L NaH_2_PO_4,_ and the flow rate was 1.0 mL/min. A calibration curve was established to quantify arginine. The experiment was performed in three biological replicates.

### NH_4_^+^ contents of fermented soybeans

Fermented soybeans (20 mg) were ground into homogenate using a mortar and pestle and dissolved in 30 mL of methanesulfonic acid (MSA) (20 mM). Then, the above solution was extracted with ultrasonication for 20 min and repeated three times. All suspensions were collected and diluted to 100 mL. The mixture was filtered with a 0.45 µm filter membrane. The NH_4_^+^ content was determined with ion chromatography according to the description in the literature [48]. Briefly, the samples were detected with an LC2010PLUS (Shimadzu, Japan) equipped with an EGC eluent generator and a DS6 conductivity detector. The chromatographic system was an IonPac CS11-HC column (4.0 × 250 mm) with an IonPac CG12A (4.0 × 50 mm) pre-column. The injection volume was set to 20 µl. The flow rate was 1.0 mL/min, and the suppression current was 59 mA. Experiments were conducted in triplicate.

## Supplementary Information

**Additional file1. Figure S1**. Data quality control; Figure S2. Expression density distribution; Figure S3. RT-qPCR of rocF; Figure S4. Construction of the homologous recombination knockout vector; Figure S5. Sequencing of BJ3-2ΔrocF; Figure S6. Fermented soybeans with BJ3-2, BJ3-2ΔrocF and BJ3-2ΔrocF+Arg at 45 °C

**Additional file2. Table S1. **Primers used in the study; Table S2. Scoring standard for sensory evaluation of BJ3-2 at 37 °C and 45 °C ;Table S3. Scoring standard for sensory evaluation of fermented soybeans by different strains. Table S4. The analysis of KEGG pathway enrichment; Table S5. Sensory evaluation of fermented soybeans by different strains

## Data Availability

The datasets supporting the conclusions of this article are included within the article.

## Abbreviations

TTMP: Tetramethylpyrazine.
SSF: Solid-state fermentation
