# Supplementary material for: rocF affects the production of tetramethylpyrazine in fermented soybeans with Bacillus subtilis BJ3-2
Source: BMC Biotechnol. 2022 Jul 4;22:18. doi: 10.1186/s12896-022-00748-4 (PMC9254598; doi:10.1186/s12896-022-00748-4)
Supplement: Supplementary file 1 — Additional file1. Figure S1. Data quality control; Figure S2. Expression density distribution; Figure S3. RT-qPCR of rocF; Figure S4. Construction of the homologous recombination knockout vector; Figure S5. Sequencing of BJ3-2ΔrocF; Figure S6. Fermented soybeans with BJ3-2, BJ3-2ΔrocF and BJ3-2ΔrocF+Arg at 45 °C [file 12896_2022_748_MOESM1_ESM.pdf]

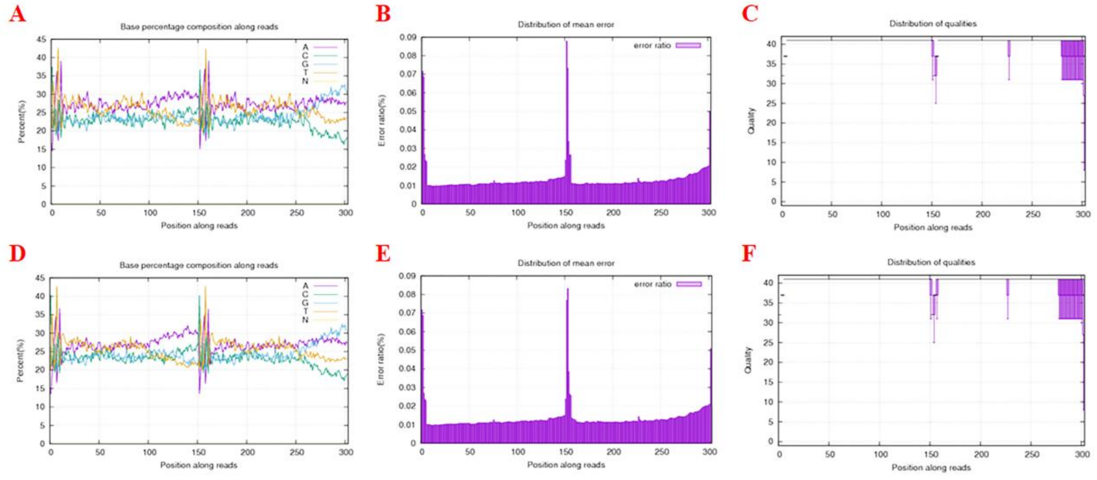

Figure S1 Data quality control

A Base composition distribution of BJ3-2 at 37℃; **B** Base error rate distribution of BJ3-2 at 37℃; **C** Base quality distribution of BJ3-2 at 37℃; **D** Base composition distribution of BJ3-2 at 45℃; E: Base error rate distribution of BJ3-2 at 45℃; **F** Base quality distribution of BJ3-2 at 45℃

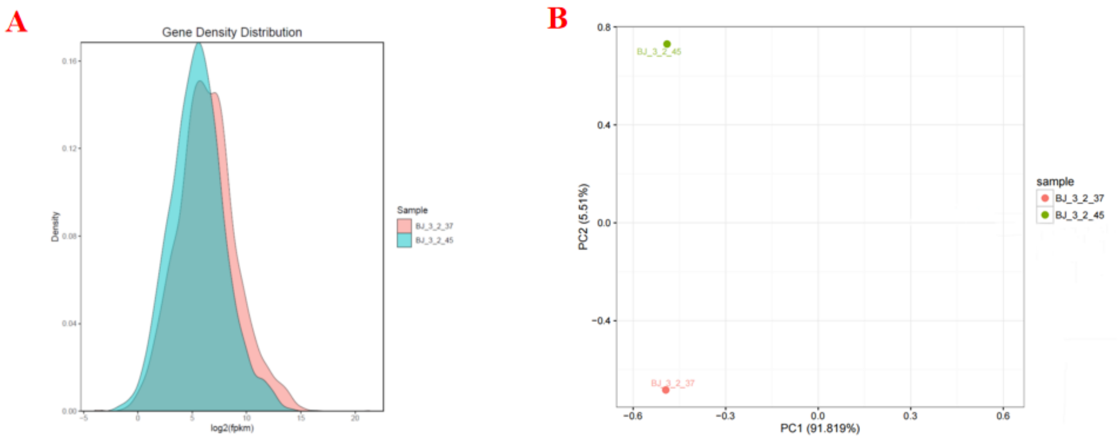

Figure S2 Expression density distribution

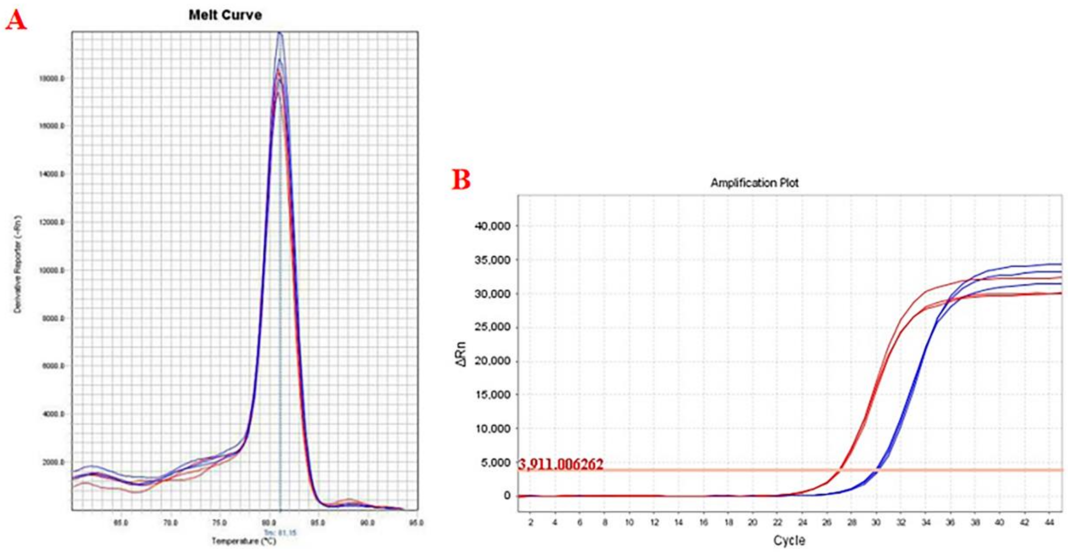

Figure S3 RT-qPCR of *rocF*

A Dissolution curve of *rocF*; **B** Amplification curve of *rocF*

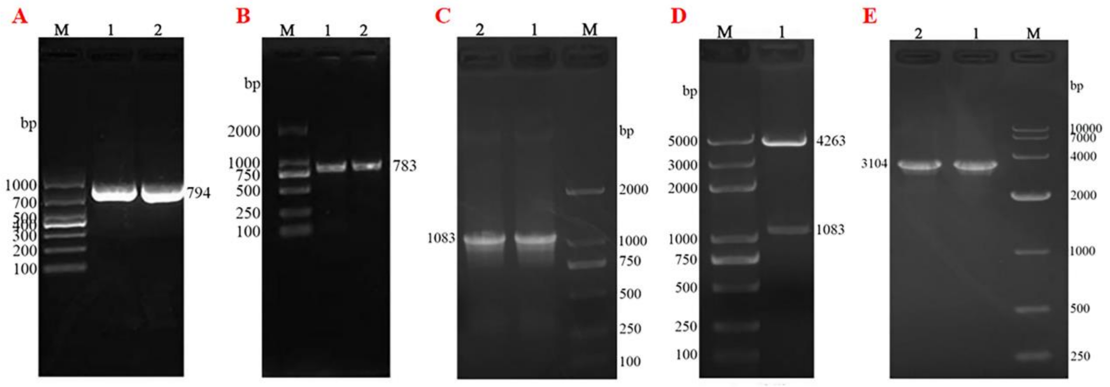

Figure S4 Construction of the homologous recombination knockout vector

A Amplification of HRarm (M: DL1000marker; lines 1-2: HRarm); **B** Amplification of HLarm (M: DL2000marker; lines 1-2: HLarm); **C** Amplification of *cm* (M: DL2000marker; lines 1-2: *cm*); **D** Double digestion of the knockout vector by *Bam*H I and *Pst* I (M: DL5000marker; line 1: double digestion); **E** Double-exchange PCR validation (M: DL10000marker; lines 1-2: transformants).

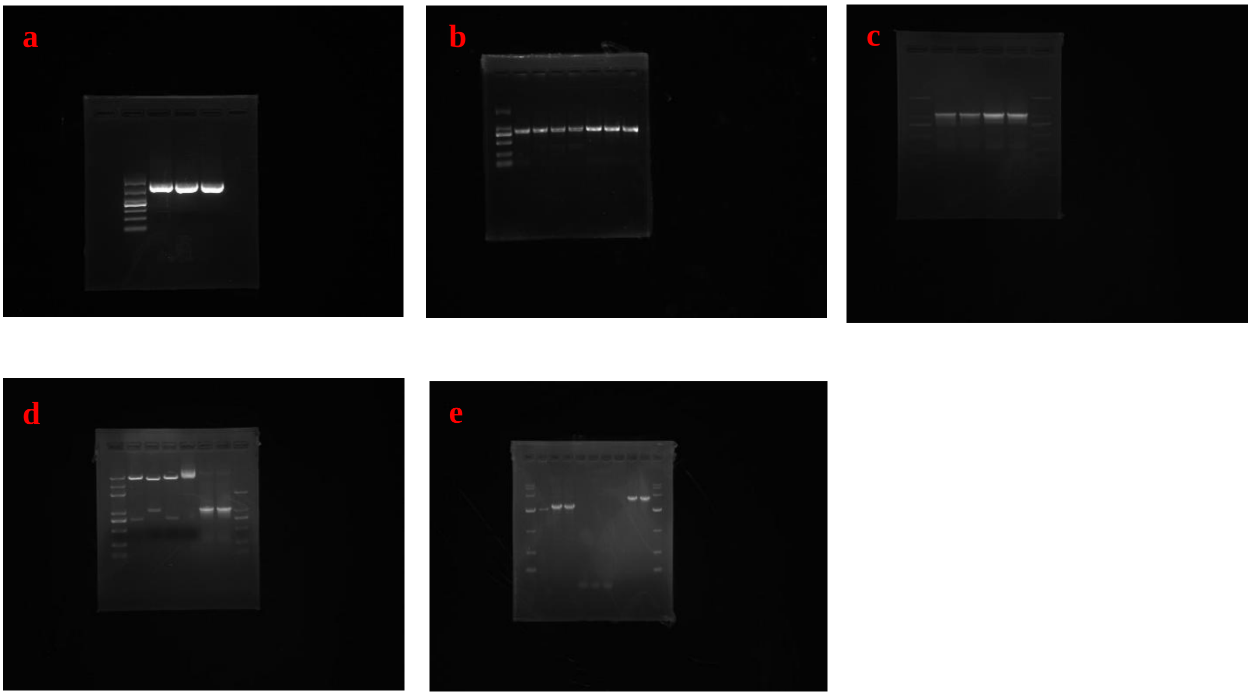

a is the original picture of Figure S4A; **b** is the original picture of Figure S4B; **c** is the original picture of Figure S4C; **d** is the original picture of Figure S4D; **e** is the original picture of Figure S4E.

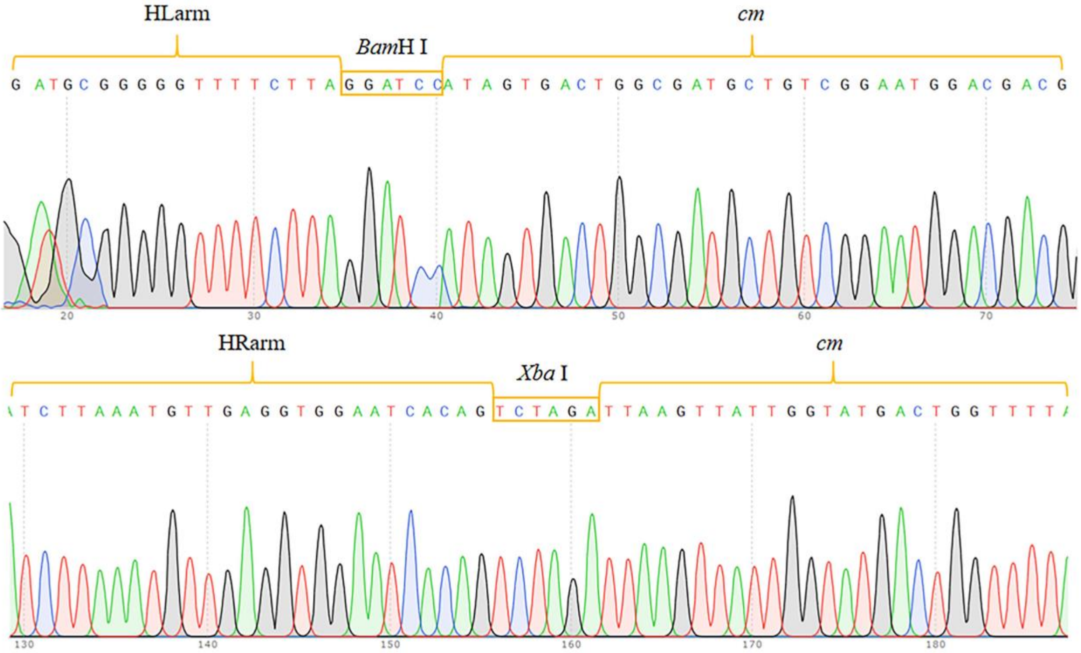

Figure S5 Sequencing of BJ3-2Δ*rocF*

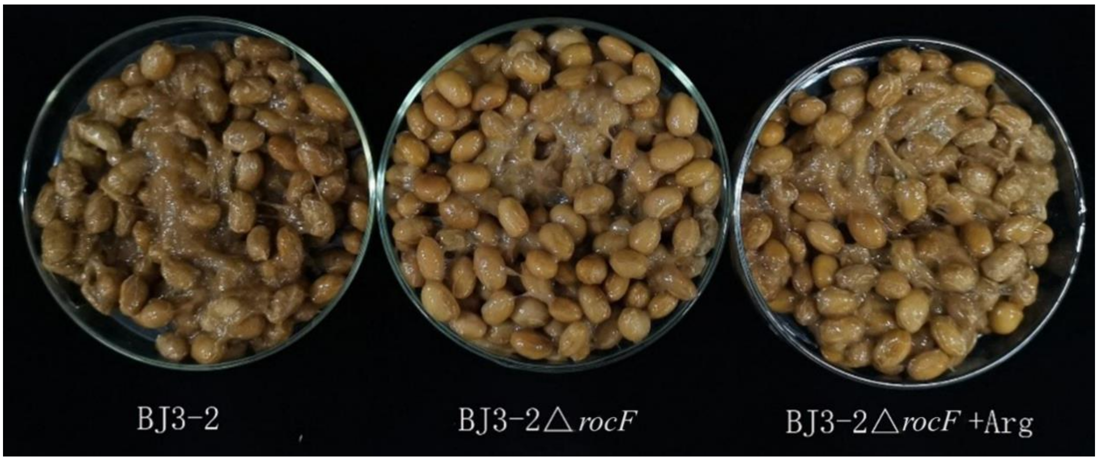

Figure S6 Fermented soybeans with BJ3-2, BJ3-2Δ*rocF* and BJ3-2Δ*rocF*+Arg at 45℃
