# Supplementary material for: rocF affects the production of tetramethylpyrazine in fermented soybeans with Bacillus subtilis BJ3-2
Source: BMC Biotechnol. 2022 Jul 4;22:18. doi: 10.1186/s12896-022-00748-4 (PMC9254598; doi:10.1186/s12896-022-00748-4)
Supplement: Supplementary file 2 — Additional file2. Table S1. Primers used in the study; Table S2. Scoring standard for sensory evaluation of BJ3-2 at 37 °C and 45 °C ;Table S3. Scoring standard for sensory evaluation of fermented soybeans by different strains. Table S4. The analysis of KEGG pathway enrichment; Table S5. Sensory evaluation of fermented soybeans by different strains [file 12896_2022_748_MOESM2_ESM.pdf]

Supplementary tables

Table S1 Primers used in the study

Name	Primer secquence(5'-3')	Enzyme site	Amplic on size
HLarm	F:CCCGAGCTCCCAATCAGAACAAGCAGTC	*Sac* I	783bp
	R:CGCGGATCCTAAGAAAACCCCCGCATCCC	*Bam*H I	
Cm	F:CGCGGATCCATAGTGACTGGCGATGCT	*Bam*H I	1083bp
	R:AACTGCAGTTAAGTTATTGGTATGACTGGTTT	*Xba* I	
HRarm	F:AACTGCAGCTGTGATTCCACCTCAACAT	*Xba* I	794bp
	R:CCCAAGCTTCGGAGAAAGTGAAGACCCT	*Hind* III	
rocF	F:TTACAGGAGCTTCTTCCCTA		891bp
	R:ATGGATAAAACGATTTCGGT		
DEDP	F:AATTATGATATTGCCATT		3104bp
	R:CTCTGATGTTTATTCTAA		
16S rRNA	F:ACTCCTACGGGAGGCAGCAG		197bp
	R:ATTACCGCGGCTGCTGG		

Table S2 Scoring standard for sensory evaluation of BJ3-2 at 37°C and 45°C

Parameters	Score	Evaluation rules
Soy sauce-like aroma	35	Good aroma (26-35), medium aroma (16-25), little aroma (1-15)
Chi-flavour	35	Good flavour (26-35), medium flavour (16-25), little flavour (1-15)
Ammonia	30	little (21-30), medium (11-20), heavy (1-10)

Table S3 Scoring standard for sensory evaluation of fermented soybeans by different strains

Parameters	Score	Evaluation rules
Colour	15	tan (8-15), dark fawn or fawn (5-7), dark brown (1-4)
Stickiness	15	Very sticky (8-15), medium sticky (5-7), slightly sticky (1-4)
Ammonia	25	little (16-25), medium (11-15), heavy (1-10)
Soy sauce-like aroma	25	Good aroma (16-25), medium aroma (11-15), little aroma (1-10)
Texture	20	Medium hard (15-20), slightly hard or slightly soft (10-14), very hard or very soft (1-9)

Table S4 The analysis of KEGG pathway enrichment

KEGG Pathway	Number	Gene
Pyrimidine metabolism	10	*carA*, *pyrAA*, *pyrC*, *pyrDI*, *pyrDII, carB (BSU11240)*, *pyrE*, *carB (BSU15520)*, *pyrF*, *pyrB*
Arginine and proline metabolism	8	*fadM*, *argF*, *argD*, *rocD*, *rocF*, *speD*, *rocG*, *rocA*
Alanine, aspartate and glutamate metabolism	7	*pyrAA*, *pyrB*, *carB*, *carA*, *rocG*, *carB*, *rocA*
Purine metabolism	6	*purD*, *purH*, *purN*, *yjmC*, *guaC*, *purS*
ABC transporters	6	*feuA*, *amyC*, *feuB*, *msmE*, *amyD*, *feuC*
Nonribosomal peptide structures	5	*ppsA*, *ppsC*, *ppsE*, *ppsB*, *ppsD*
Nitrogen metabolism	4	*narJ*, *narI*, *narG*, *rocG*
Biosynthesis of amino acids	4	*argD*, *rocF*, *argF*, *gapB*
Phosphotransferase system (PTS)	3	*licC*, *nagP*, *licA*
Glycolysis / Gluconeogenesis	3	*pckA*, *licH*, *gapB*
Two-component system	3	*narJ*, *narI*, *narG*
Carbon fixation in photosynthetic organisms	2	*pckA*, *gapB*
One carbon pool by folate	2	*purH*, *purN*
Sulfur metabolism	2	*yvgQ*, *cysJ*
Histidine metabolism	2	*hutG*, *hutI*
Pentose and glucuronate interconversions	2	*uxuA*, *uxaC*
Glycine, serine and threonine metabolism	2	*gbsA*, *gbsB*
Amino sugar and nucleotide sugar metabolism	2	*nagP*, *nagBB*
Carbon metabolism	2	*pckA*, *gapB*
Taurine and hypotaurine metabolism	1	*rocG*
HIF-1 signaling pathway	1	*gapB*
Glyoxylate and dicarboxylate metabolism	1	*oxdC*
Citrate cycle (TCA cycle)	1	*pckA*
Fructose and mannose metabolism	1	*gmuG*
2-Oxocarboxylic acid metabolism	1	*argD*
Cysteine and methionine metabolism	1	*speD*
Pyruvate metabolism	1	*pckA*

Table S5 Sensory evaluation of fermented soybeans by different strains

Strain	Appearance(30)		Aroma (50)		Texture (20)	Totalscore
	Colour (15)	Stickiness (15)	Ammonia (25)	Soy sauce-like aroma (25)		
BJ3-2 45℃	7	12	15	15	17	66
BJ3-2 Δ*rocF* 45℃	10	14	22	24	18	88
